# Yellow Fever 17DD Vaccine Virus Infection Causes Detectable Changes in Chicken Embryos

**DOI:** 10.1371/journal.pntd.0004064

**Published:** 2015-09-15

**Authors:** Pedro Paulo de Abreu Manso, Barbara C. E. P. Dias de Oliveira, Patrícia Carvalho de Sequeira, Yuli Rodrigues Maia de Souza, Jessica Maria dos Santos Ferro, Igor José da Silva, Luzia Fátima Gonçalves Caputo, Priscila Tavares Guedes, Alexandre Araujo Cunha dos Santos, Marcos da Silva Freire, Myrna Cristina Bonaldo, Marcelo Pelajo-Machado

**Affiliations:** 1 Laboratório de Patologia, Instituto Oswaldo Cruz, FIOCRUZ, Rio de Janeiro, Brazil; 2 Laboratório de Biologia Molecular de Flavivírus, Instituto Oswaldo Cruz, FIOCRUZ, Rio de Janeiro, Brazil; 3 Universidade Federal do Estado do Rio de Janeiro, UNIRIO, Rio de Janeiro, Brazil; 4 Instituto de Tecnologia em Imunobiológicos, Fundação Oswaldo Cruz, Rio de Janeiro, Brazil; Baylor College of Medicine, UNITED STATES

## Abstract

The yellow fever (YF) 17D vaccine is one of the most effective human vaccines ever created. The YF vaccine has been produced since 1937 in embryonated chicken eggs inoculated with the YF 17D virus. Yet, little information is available about the infection mechanism of YF 17DD virus in this biological model. To better understand this mechanism, we infected embryos of *Gallus gallus domesticus* and analyzed their histopathology after 72 hours of YF infection. Some embryos showed few apoptotic bodies in infected tissues, suggesting mild focal infection processes. Confocal and super-resolution microscopic analysis allowed us to identify as targets of viral infection: skeletal muscle cells, cardiomyocytes, nervous system cells, renal tubular epithelium, lung parenchyma, and fibroblasts associated with connective tissue in the perichondrium and dermis. The virus replication was heaviest in muscle tissues. In all of these specimens, RT-PCR methods confirmed the presence of replicative intermediate and genomic YF RNA. This clearer characterization of cell targets in chicken embryos paves the way for future development of a new YF vaccine based on a new cell culture system.

## Author Summary

Since 1937, the vaccine against yellow fever has been produced in chicken embryos without any critical modification. Despite this highly available and effective vaccine, yellow fever remains an important cause of morbidity and mortality in tropical regions of Africa and South America, mainly by maintaining the sylvatic cycle (in which mosquitoes transmit the virus from non-human primates to people who visit or work in the jungle). To our knowledge, the present study offers the first clear elucidation of cells and tissues associated with the biosynthesis of the17DD yellow fever virus in chicken embryos. We detected that the virus causes only mild lesions in the embryos, but affects different cells and tissues, including muscles, cells in the heart muscles and in the nervous system, certain tissues in the kidneys and lungs, and collagen-producing cells that exist in connective tissues in the cartilage and skin. Identifying these cells may help scientists develop a cell-culture-based vaccine with lower chick protein content. This understanding is important because recent data indicate that the YF virus 17DD may serve as a platform to produce other new recombinant vaccines.

## Introduction

Yellow fever (YF) is a viral disease associated with a flavivirus infection that affects individuals in the tropical regions of South America and Sub-Saharan Africa. The course of the disease may be mild, subclinical, or abortive (with flu-like symptoms), or severe. The severe form is pansystemic: it affects the liver, kidneys, and myocardium, and includes hemorrhage and shock. Over 50% of patients with severe YF die [1,2].

Studies have described the pathology and pathogenesis of YF in fatal human cases, and in experimental infections of non-human primates, golden hamsters, and mice [3–7]. However, all these models can only provide information on YF pathology of fatal cases. There are no available models for the study of moderate, mild, and subclinical forms of YF [6].

Natural infection happens when an infected mosquito bites a person and inoculates the virus into the dermis of the host. The inoculated virus at first infects dendritic cells in the skin, which are also susceptible to virus infection *in vitro* and likely play an important role in infection by other flavivirus such as Dengue [3,8–10]. Then, lymphatic vessels drain these cells to lymph nodes, where the virus is replicated and released into the bloodstream, causing the first viremia [3,8,11–13]. Once in hematogenous route, the virus can affect the liver, kidneys, heart, spleen, and other organs, infecting mainly hepatocytes, Kupffer cells, cardiomyocytes, and epithelial cells of the renal tubule [4,11]. Morphologically, infection of these cells may generate acidophilic corpuscles (in the liver called Councilman corpuscles or Rocha-Lima lesions), microsteatosis, and apoptotic bodies [14]. The local inflammatory response, when compared to organ injuries, is not significant. Minimal or moderate infiltrates are observed in the portal space, with lymphocytes and monocytes predominating [11].

There is no antiviral treatment for this disease, and the only way to control it is to preventively vaccinate populations living in at-risk areas [1,2]. The YF 17D vaccine effectively protects over 98% of immunized individuals for at least 20–35 years and probably for life, following vaccination [15]. Despite the wide availability of this vaccine, YF continues to cause morbidity and mortality in tropical regions of Africa and South America [16,17]. In these regions, both persons residing in endemic or epizootic areas and unvaccinated travelers are at risk of infection.

Complicating matters, the vaccine is also contraindicated or demands precaution for a number of patients, including: those with allergies to eggs or other vaccine components (which are difficult to identify due to trade secret laws), women who are pregnant or breastfeeding, children less than six months old, individuals older than 60, transplant recipients, patients with AIDS, patients presenting primary immunodeficiency, and immunosuppressed patients with cancer or thymic diseases [18]. Although the YF vaccine is generally very safe and effective, viscerotropic and neurotropic vaccine-associated diseases have occurred, especially in patients with immunodeficiency or the elderly [19]. These vaccine-associated diseases can kill up to 65% of affected patients due to the lack of available treatment. The physiopathology of these unexpected reactions to the vaccine remains unclear [8].

The YF vaccine was developed from the sample Asibi, isolated from a patient named Asibi, a mild case of YF, who survived to this infection in Ghana in 1927 [20]. The 17D strain became attenuated for humans after serial passages in chicken and mouse tissue cultures. Two main sub-strains were independently derived from 17D, called 17DD and 17D-204 [21]. The 17DD strain was first used in Brazil in 1937, and approximately 500 million doses have been administered worldwide since the seed lot system was introduced in 1945 [22]. The vaccine is still produced today by inoculating 17DD YF virus in ninth day embryonated chicken eggs free of specific pathogens (SPF), which are processed 72 hours later according to the standards set by the World Health Organization [22]. Although chicken embryos have been used since 1937 to produce the YF virus, the histopathology of this infection is scarcely studied and the molecular mechanisms that regulate the viral infection in this biological system are still not well understood. For instance, it is not known which cells biosynthesize 17DD viral particles in infected chicken embryos. This knowledge would be of great importance, since these virus-producing cells would be the initial candidates for the future development of a YF vaccine based on a cell culture system. In this study, our aim was to establish which tissues and cells are responsible for YF viral production, and to characterize the 17DD YF virus infection in *Gallus gallus domesticus* embryos in terms of the histopathological changes mediated by the viral infection in conditions similar to those used in the production of YF vaccine. Our data contribute to the literature of the histopathology of the chicken biological system, and help advance the knowledge of the histopathological peculiarities involved in the pathogenesis of YF. Identifying these competent cells could help researchers develop a vaccine with lower non-viral protein content, based on a cell culture system. A vaccine with lower chick protein content has the potential to reduce allergic and other adverse reactions, and therefore to help at least a subset of the population for whom the traditional vaccine is counter-indicated.

## Materials and Methods

### Biological System

Specific pathogens-free (SPF) fertilized White Leghorn chicken eggs (*Gallus gallus domesticus*; Linnaeus, 1758) were obtained from the YF vaccine production unit (FIOCRUZ). Eggs were infected in the yolk sac with 17DD EPlow virus seed lot (1–5 x 10^3^ PFU per inoculum) in the ninth day of development [21,23,24]. Eggs were kept in an IP70 brooder (Premium Ecologica, Brazil) with controlled temperature at 37.5°C, and 55% relative air humidity. As negative controls, embryos kept under the same conditions were inoculated with water for injection. For all analyses, embryos were collected at 72 hours post infection (12 days of development). The mean of the yield of virus from infected eggs, titrated by plaque formation assays (PFU), was 6,74 log10 PFU/ml (ranging from 6,24 to 6,97 log10 PFU/ml).

### Ethics Statement

This work was conducted with fertilized White Leghorn chicken eggs (*Gallus gallus domesticus*) with nine to twelve days of development, obtained from Instituto de Tecnologia em Imunobiológicos (Fiocruz, Rio de Janeiro, Brazil). All experiments are in accordance to the yellow fever vaccine production protocol, which has been applied since 1937, when the vaccine production started at the mentioned unit, under ethical approval of Fiocruz.

### Histopathological Analysis

Chicken embryos, yolk sacs, and chorioallantoic membranes were collected and dissected. Membranes were cleaved in regions defined by quadrants. For each embryo, the head, whole wings, and whole legs were separated from the trunk, and subsequently cleaved. The trunks were transversely sectioned into subsequent samples of about 3 mm. All fragments were fixed in Carson`s formalin-Millonig for 48 hours at room temperature [25], and processed according to standard histological techniques for paraffin embedding. Sections (5 μm thick) were stained with hematoxylin-eosin [26]. The slides were analyzed in an Axiovert Z1 microscope (Carl Zeiss, Germany), and the images were acquired with an mRC5 Axiocam digital camera (Carl Zeiss, Germany).

### Immunofluorescence Assay

Twenty-four hours after they were obtained, sections of all paraffin blocks from infected and control animals were de-waxed, dehydrated, and washed in PBS. Antigenic retrieval was carried out in 0.01 M citrate buffer pH 6.0 in Pascal chamber (Dako, USA), according to the manufacturer’s recommendations. The sections were incubated with a blocking solution (2% skimmed milk, 2.5% bovine serum albumin, and 8% fetal bovine serum in the same buffer) in a humid chamber for 30 minutes at room temperature, and kept overnight with an anti-YF virus antibody at 4°C. Two polyclonal mouse antibodies directed against YF virus were used (Yellow Fever virus hyperimmune serum–Evandro Chagas Institute, and Yellow Fever 17D hyperimmune ascitic fluid cod. V525701562 –NIH). There was no difference between the anti-YF antibodies: both recognized the same set of cells, and did not react in negative controls. AlexaFluor 488-conjugated goat anti-mouse secondary antibody in 1:750 dilution (cat. A11001, Life Technologies, USA) was applied at 37°C for 1 hour followed by counterstaining with 1:5,000 DAPI (cat. 03571, Molecular Probes, USA). Double staining used an anti-desmin antibody in 1:100 dilution (cat. RB-9014, Thermo Scientific, USA) applied at 37°C for 1 hour followed by an AlexaFluor 546-conjugated goat anti-Rabbit secondary antibody in 1:750 dilution (cat. A11010, Life Technologies, USA). Negative controls were performed by duplicating each sample and omitting treatment with the primary antibodies, so that any reactions resulting from the secondary antibodies or reagents employed in the analyses could be adequately traced. Sections were mounted in ProlongGold (cat. P36934, Life Technologies, USA) and analyzed in an LSM 710 or LSM 880 Airyscan confocal microscope or an ELYRA SR-SIM microscope (Carl Zeiss, Germany).

### Nested-PCR

RNA samples were extracted from formalin-fixed, paraffin-embedded tissue from the same blocks used in immunofluorescence analysis, which were either positive or negative to 17DD virus. Two 10μm thick sections were put in a microtube and submitted to PureLink FFPE Kit (cat. 45–7015, Life Technologies, USA), according to the manufacturer's recommendation for RNA extraction. RNA samples eluted after the procedure were amplified by Reverse Transcription-PCR carried out with Thermoscript RT-PCR kit (cat. 11146016, Life Technologies, USA), with universal Flavivirus primers described by Tanaka [27] (YF1–5`GGTCTCCTCTAACCTCTAG 3`and YF3–5`GAGTGGATGACCACGGAAGACATGC 3`). After that, a second amplification was carried out with internal primers designed by our group (YF2–5`CGAGTTTTGCCACTGCTAAGCT 3`and YF4–5`TAGACCCCGTCTTTCTACCACC 3`). Two different reactions were performed using specific primers in RT-PCR using forward and reversed YF-1 and YF-3 primers to detect the genomic RNA and the replicative intermediate. After amplification, the nested-PCR product was sequenced in DNA Analyzer ABI 3730 (Applied Biosystems, USA), and aligned to the YF Virus 17DD genomic sequence (GenBank U17066.1) using ClustalW2 [28].

## Results

### Histopathological Changes Associated with YF Infection

**Fig 1:**
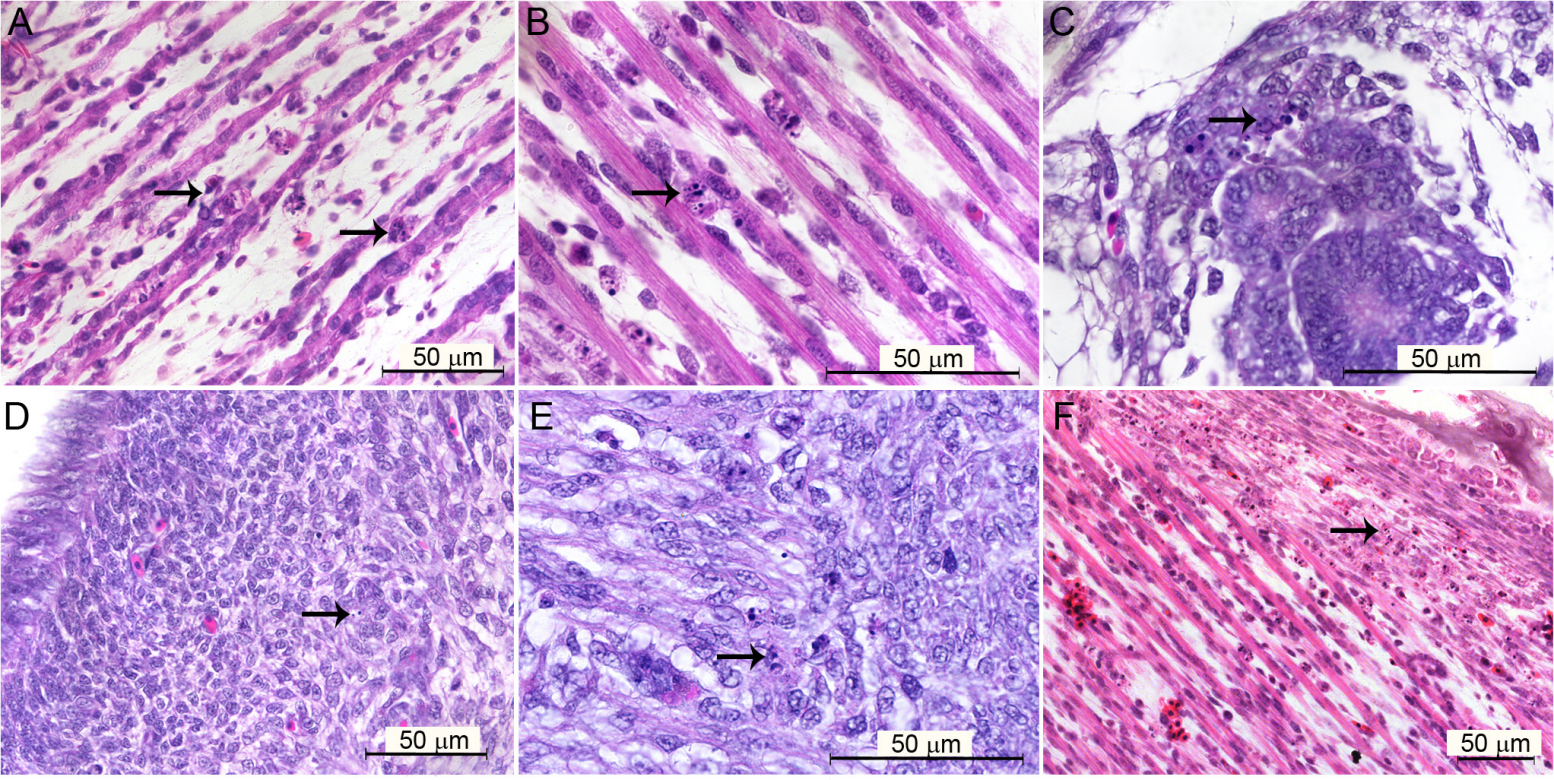
Brightfield microscopy of tissues of *Gallus gallus domesticus* 72 hpi with yellow fever 17DD virus. (A) Apoptotic bodies in skeletal muscular tissue; (B) detail of karyorrhexis in muscular bundles; (C) apoptotic bodies in tubular epithelium; (D) apoptotic bodies in muscular region of gizzard; (E) detail of apoptotic bodies in the muscular layer of the gizzard; (F) apoptotic bodies in fibroblastoid cells of perichondrium. Apoptotic nuclei are indicated by black arrows (→). Hematoxylin and Eosin stain.

Different embryonic tissues were stained with hematoxylin and eosin to investigate the histopathological alterations caused by the YF 17DD at 72 hours post-infection. In most cases, few differences in controls and infected embryos were observed. However, some embryos had mild focal reactions to infection, expressed by apoptotic bodies in infected tissues, including muscular tissue (Fig 1A and 1B), renal tubular epithelium (Fig 1C), parenchyma of the gizzard (Fig 1D and 1E), and fibroblastoid cells in perichondrium (Fig 1F).

In addition, both infected and control embryos showed extensive areas of hematopoiesis in the yolk sac, which presented scattered blastoid cells in perivascular sheaths. The bone marrow of some embryos (infected and controls) was already formed and functional. In this embryonic stage, gastric and respiratory epithelia were developing and showed heterogeneous cellular morphology, and sometimes apoptotic cells.

### Detection of YF Infection Sites by Immunofluorescence Microscopy

**Fig 2:**
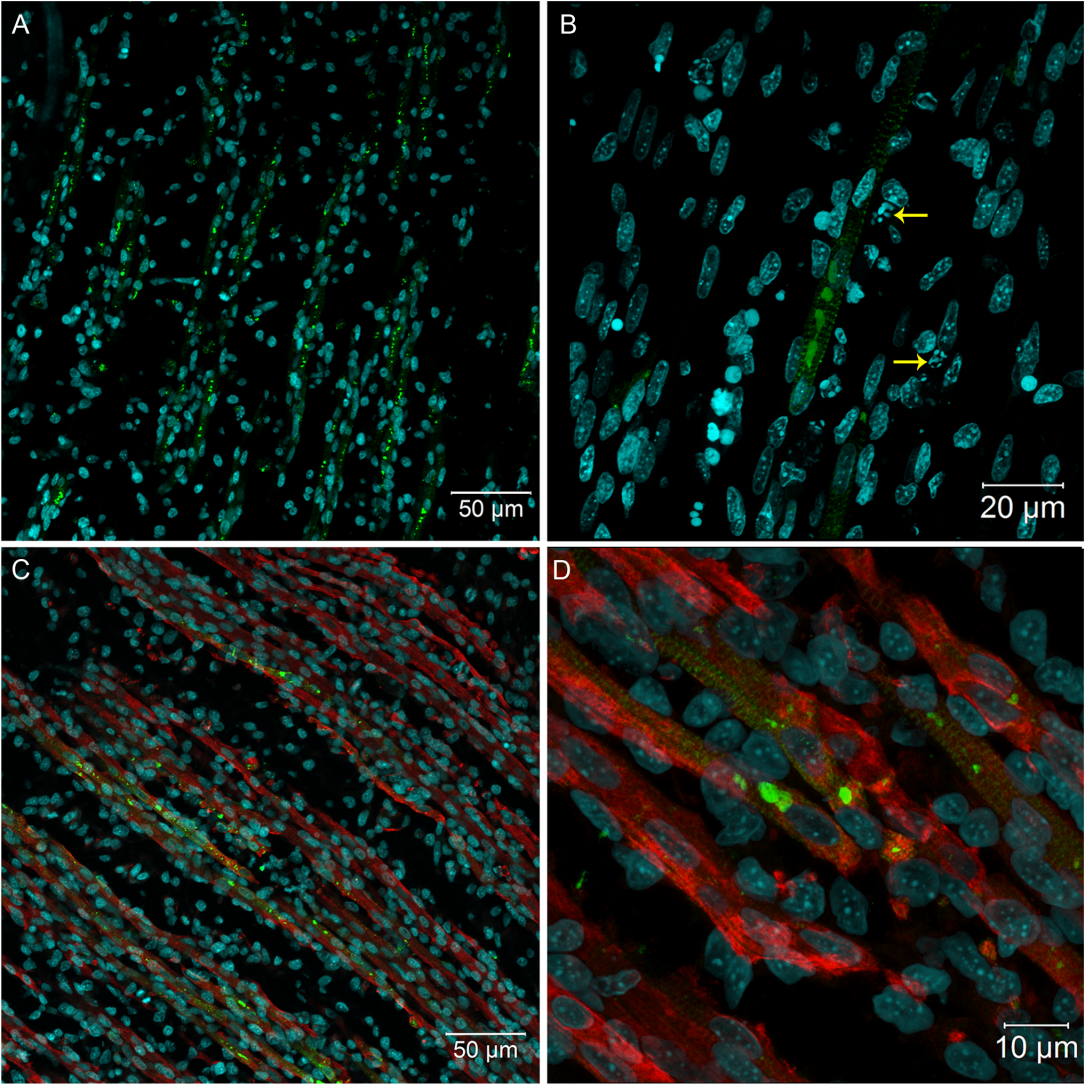
Immunofluorescence in skeletal muscle tissue of chicken embryos at 72 hpi with 17DD virus. Polyclonal antibodies directed against the yellow fever virus were used to immunostain virus proteins in: (A) skeletal muscle cell bundles; (B) skeletal muscle cells showing perinuclear thickening and presenting an intense labeling in sarcoplasmic reticulum following the striations of the cytoskeleton–yellow arrows (→) show pyknosis and karyorrhexis close to infected cells; and (C) skeletal muscle cells evidenced by desmin antibody and showing the virus infection of the muscular bundles. (D) Detail of the infected muscular bundles, showing the perinuclear positivity together with striations. Yellow fever virus staining in green, nuclei stained with DAPI in blue and desmin in red.

**Fig 3:**
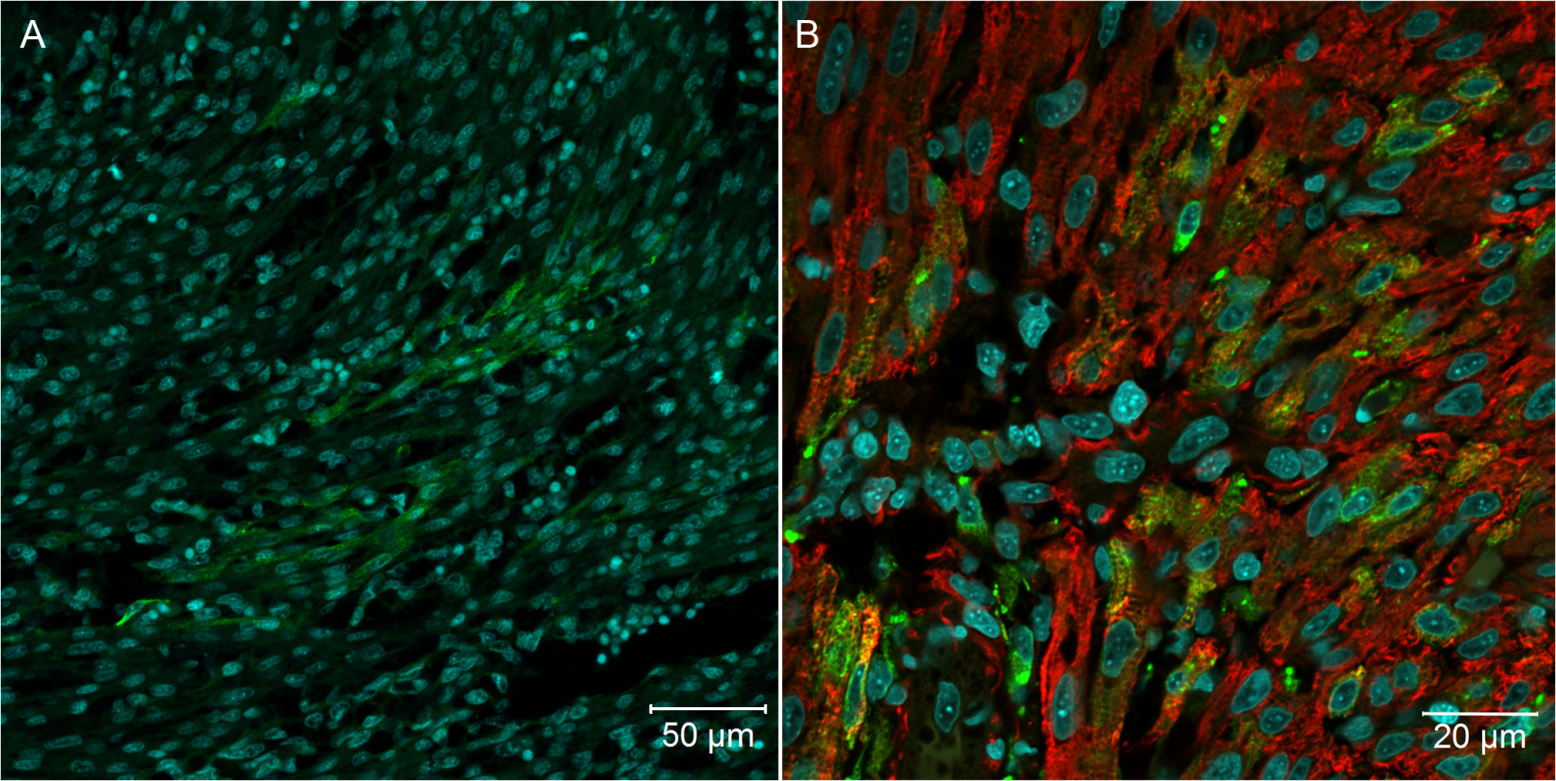
Heart muscular tissue of *Gallus gallus domesticus* at 72 hpi with yellow fever 17DD virus. (A) Infected heart muscle cells; (B) desmin positive heart muscle cells showing perinuclear virus protein distribution and striated pattern compatible with sarcoplasmic virus protein distribution. Yellow fever viral antigen detection in green, nuclei stained with DAPI in blue and desmin in red.

**Fig 4:**
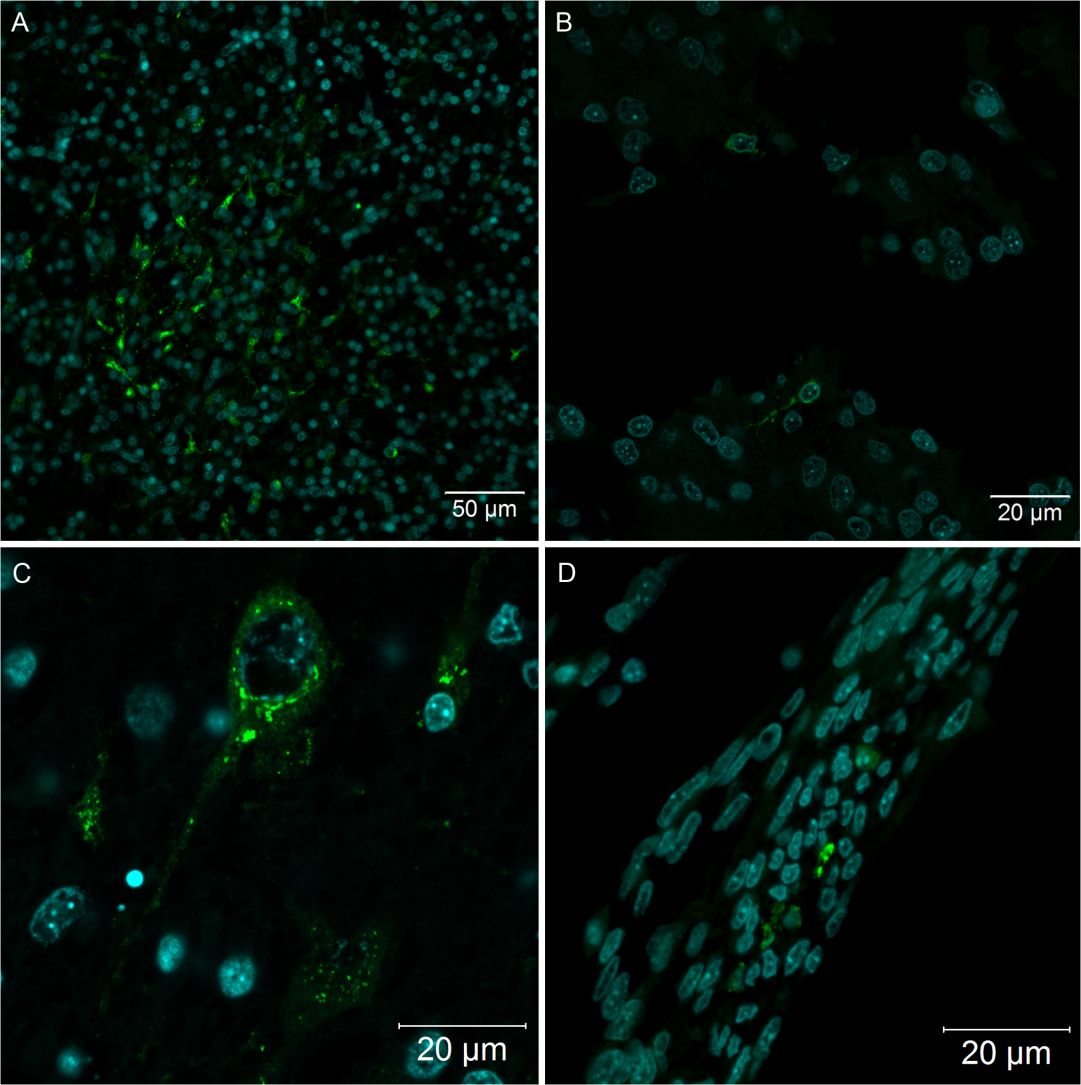
Nervous system of *Gallus gallus domesticus* at 72 hpi with yellow fever 17DD virus. (A) Brain section presenting some infected neurons and glial cells; (B) spinal cord infected neurons; (C) one neuron of the brain showing perinuclear thickening and vesicles dispersed throughout the cytoplasm; (D) infected fibroblastoid cells along the meninges. Yellow fever virus protein detection in green and nuclei stained with DAPI in blue.

**Fig 5:**
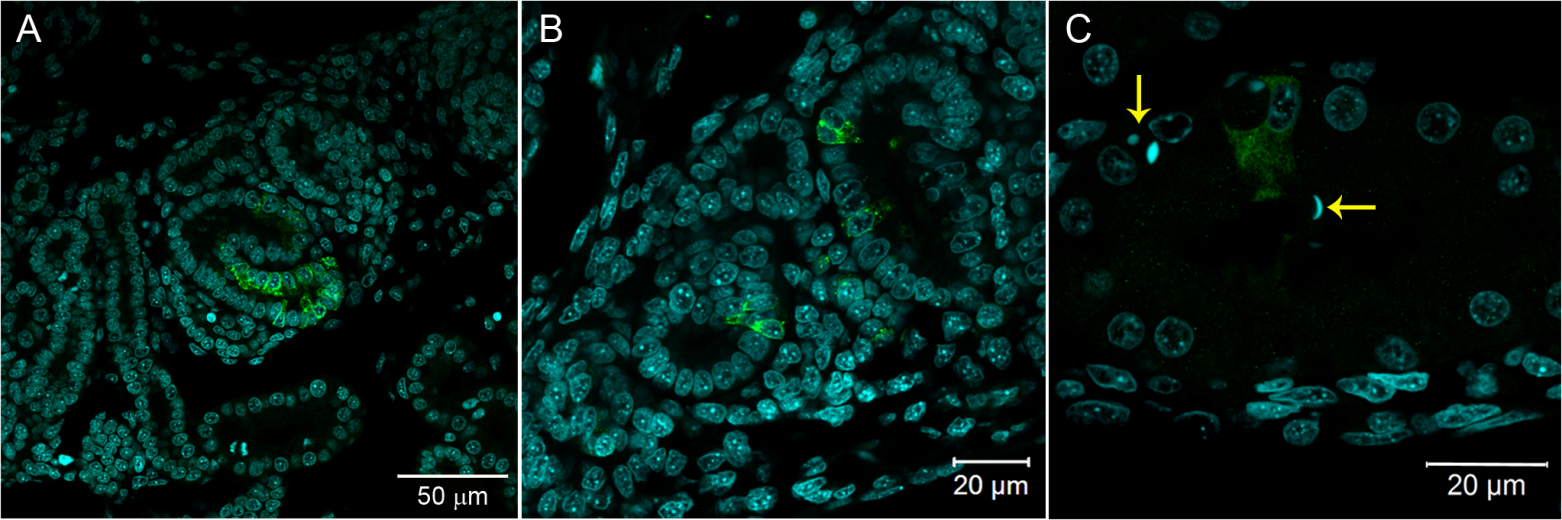
Tubular epithelial cells in *Gallus gallus domesticus* at 72 hpi with yellow fever virus. (A, B) Infected kidney tubular epithelium cells; (C) fluorescent vesicles inside tubular kidney cells, suggesting virus excretion to the lumen of the tubules–pyknotic nuclei are indicated by yellow arrows (→). Yellow fever virus detection in green and nuclei stained with DAPI in blue.

**Fig 6:**
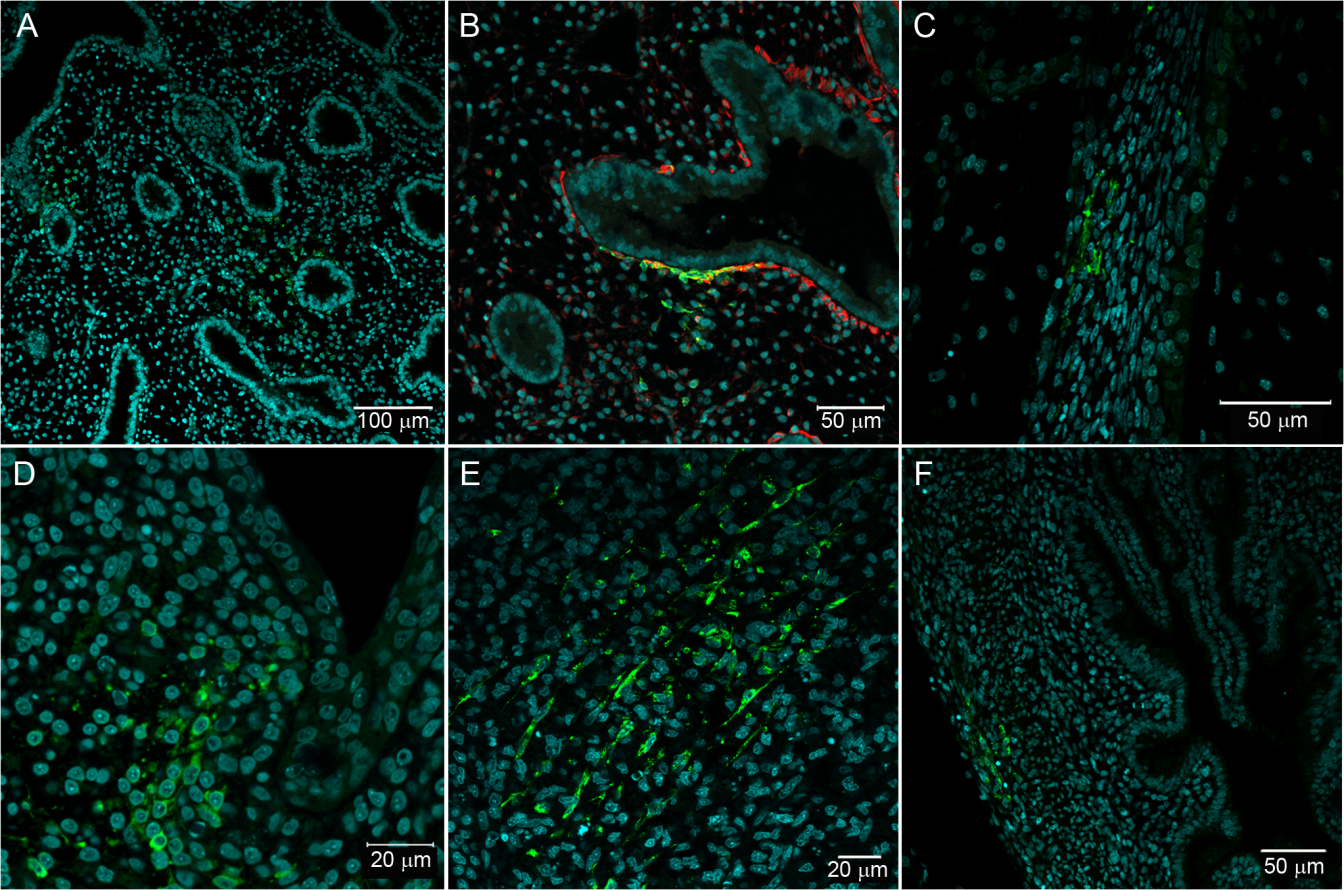
Fibroblastoid cells in different sites of *Gallus gallus domesticus* at 72 hpi with 17DD virus. (A) Parenchyma lung cells surrounding the parabronchi epithelium; (B) detail of parenchymal positive cells with desmin expression; (C) infected fibroblastoid cells along the perichondrium; (D) infected cell cluster in subepithelial connective tissue; (E) infected cells in the muscular layer of the gizzard; (F) infected cells in the muscular layer of the yolk stalk. Yellow fever virus proteins immunostained in green, nuclei stained with DAPI in blue and desmin in red.

Because our histopathological data revealed mild changes that could be associated with YF viral infection, we decided to analyze several tissues of YF-infected embryos using more sensitive techniques: specifically, confocal and super-resolution immunofluorescence microscopy. Using anti-YF antibodies, we clearly identified viral proteins in skeletal muscle tissue (Fig 2), cardiomyocytes (Fig 3), neurons and glial cells in the brain (Fig 4A and 4C), spinal cord neurons (Fig 4B), renal tubular epithelium (Fig 5), lung parenchyma (Fig 6A and 6B), and fibroblasts associated with connective tissue in the perichondrium (Fig 6C) and dermis (Fig 6D).

**Fig 7:**
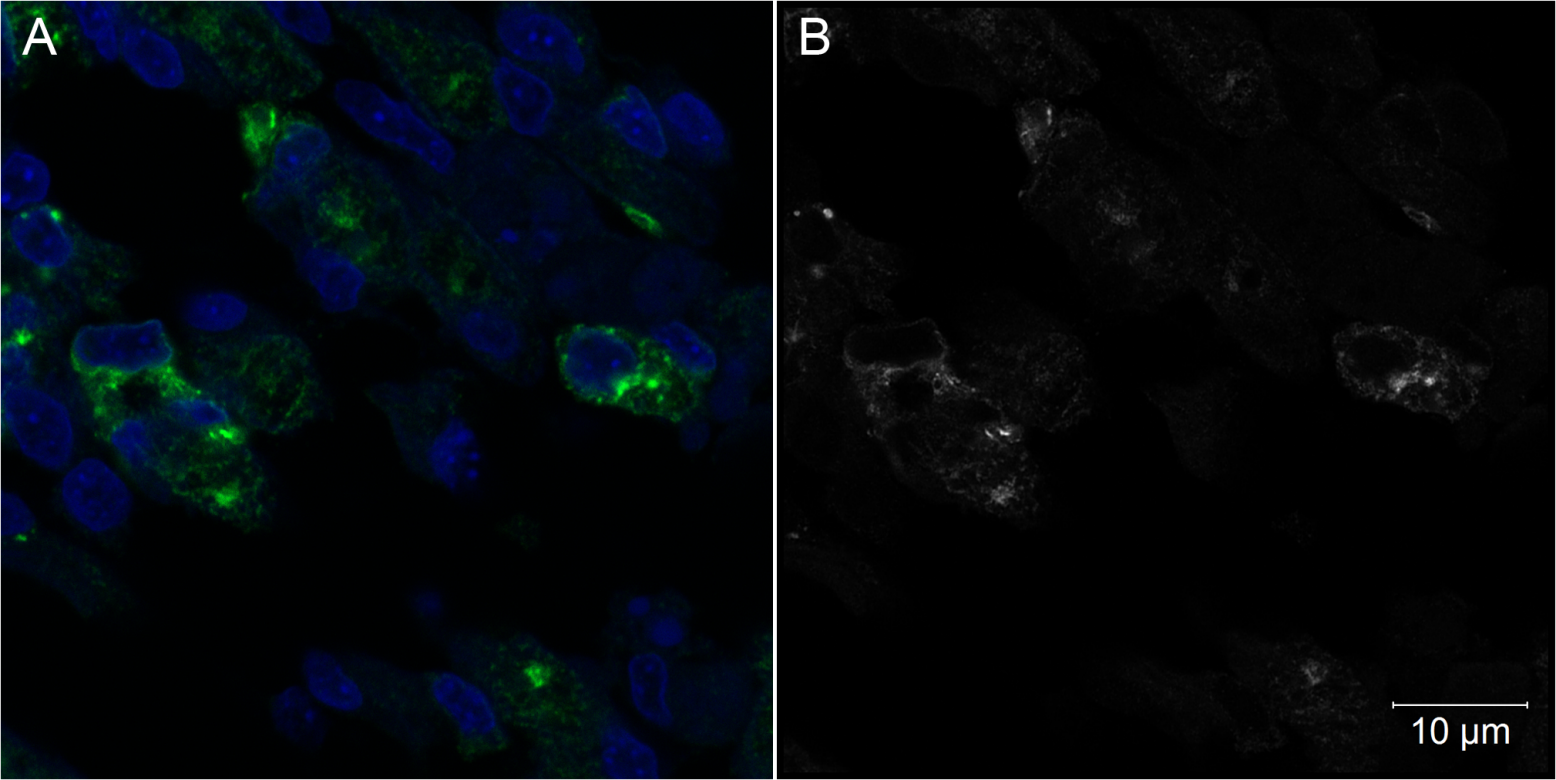
Intracellular aspects of muscle cells of *Gallus gallus* at 72 hpi with 17DD virus. (A) Skeletal muscle cells detected by confocal microscopic analysis; (B) Airyscan super-resolution microscopy of the same field of view in a 0.16 μm optical slice showing a vesicular pattern of virus protein expression. Yellow fever virus proteins detected in green (A) or in white (B), nuclei stained with DAPI in blue (A).

**Fig 8:**
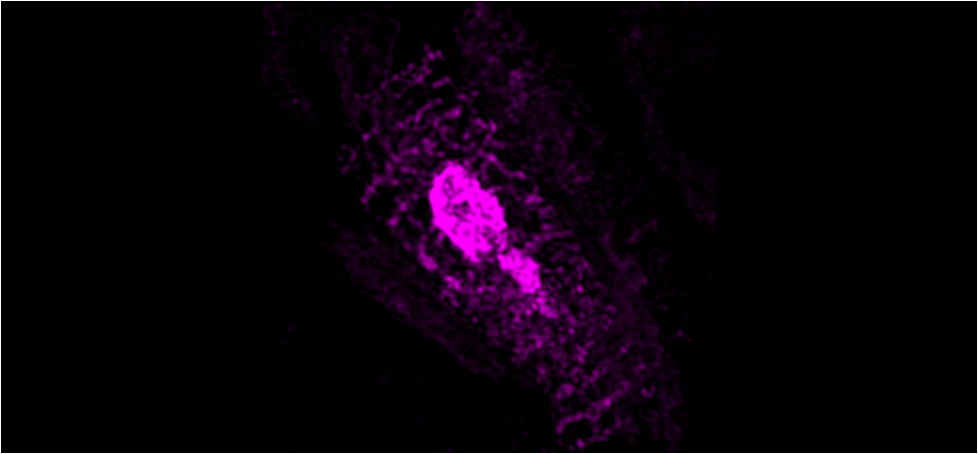
SR-SIM microscopy of chicken muscle cells at 72 hpi with 17DD virus. The virus proteins follow the striations in a localization compatible with the sarcoplasmatic reticulum. In the center it is possible to see an agglomeration of viral proteins probably inside the endoplasmic reticulum.

We also found either isolated and intense positive cells (or small cell clusters) contrasting with extensive negative cell areas in all studied tissues. These YF-infected cells had a characteristic staining pattern with perinuclear and hypertrophied endoplasmic reticulum, and vesicles dispersed throughout the cytoplasm (Figs 2B, 2D and 4C). When analyzed by super-resolution microscopy with 0.16 μm optical slice, the endoplasm reticulum and vesicles carrying viral proteins were more evident (Figs 7 and 8).

The viral antigen was found in skeletal muscle cells throughout the body of the embryos. The infection affected the entire length of some muscle bundles (Fig 2A), where sometimes viral antigen detection followed the striations of the cytoskeleton (Figs 2B and 8). In some regions, it was possible to identify cells with pyknosis and karyorrhexis figures close to infected cells (Fig 2B). At least at 72 hours post infection, the striate muscle tissue seemed to be an important site of production of the YF 17DD virus in embryos of *Gallus gallus domesticus*. The infection affected muscle bundles of the head, trunks, legs, and wings of the chicken embryos. However, the distribution of infected cells was similar across these regions. An anti-desmin antibody was then used to improve the identification of the muscle fibers, and showed a strong positive result in these cells (Fig 2C and 2D).

Virus proteins forming positive clusters were found in focal areas of the heart (Fig 3). Double staining with the desmin antibody revealed that only cardiomyocytes were infected (Fig 3B).

In the brain (Fig 4A and 4C), the number and localization of positive cells varied among embryos, with the cerebellum showing the highest number of positive cells. In some embryos, isolated neurons in the spinal cord were positive for virus production (Fig 4B), and we identified positive cells with a fibroblastoid pattern in the meninges (Fig 4D).

Viral antigens were identified in some epithelial cells of the kidney tubules, with an intense cytoplasmic pattern (Fig 5). The infection occurred in one or more cells per tubule section. Often, the presence of fluorescent vesicles suggests virus excretion to the lumen of the tubules (Fig 5B and 5C). On the other hand, Bowman's capsule was always negative to virus protein labeling.

In some embryos, virus proteins were observed in mesenchymal cells of the lung parenchyma surrounding the bronchi and parabronchi (Fig 6A). These cells were strongly positive for desmin (Fig 6B). The lung epithelium of all animals was free of viral antigens.

Besides the positive organs and cells, we also detected viral protein labeling in fibroblastoid cells along the bodies of some animals (in isolated areas of the subepithelial connective tissue) (Fig 6D). A few animals also had positive fibroblastoid cells in the muscular gizzard region (Fig 6E).

Using immunofluorescence, we were able to detect the virus in cells of the muscular layer of yolk stalk of one animal (Fig 6F). All cells in the vitelline and in the chorioallantoic membranes were negative. Remarkably, the livers of chicken embryos were always free of viral antigens, suggesting that at least in this embryonic stage and time of infection, the liver of *Gallus gallus domesticus* may be impervious to YF infection.

### Detection of Genomic and Intermediated Replicative YF RNA from FFPE Samples by Nested-PCR Assays

**Fig 9:**
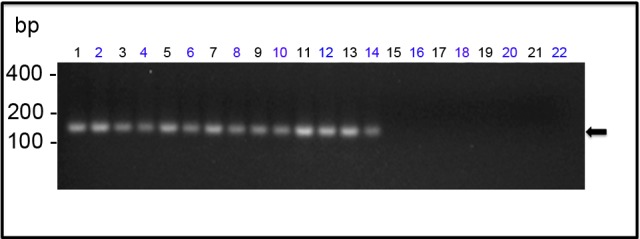
Detection of viral genomic RNA in YF 17DD-infected chicken embryos. The amplicons generated by Nested-PCR were analyzed by 2% agarose gel electrophoresis. The lanes correspond to the following specimens: (1) and (2)—head; (3) and (4)—legs; (5) and (6)—wings; from (7) to (14)—trunks; (15) and (16)—vitelline membrane; (17) and (18)—chorioallantoic membrane; from (19) to (22)—negative control (water-inoculated animals). Even-numbered lanes indicate samples submitted to amplification of genomic RNA whereas odd-numbered lanes indicate samples submitted to amplification of the replicative intermediate RNA. The molecular length markers are indicated on the left of the figure. The black arrow indicates the 156bp amplicon obtained from the amplification of YF 17D RNA.

We confirmed the pattern of YF17DD virus distribution in different tissues using RNA extraction from formalin-fixed, paraffin-embedded tissues (FFPE) followed by viral RNA amplification by Nested-PCR. This technique showed itself to be sensitive enough to amplify RNA viral fragments, and it was possible to detect genomic viral RNA in all immunofluorescence positive blocks. We were able to detect amplicons of 156 bp (the expected size products corresponding to the YF genome position from 10556 to 10711) in specimens from the legs, wings, head, and trunks of all YF 17DD-infected embryos. Fragments from the chorioallantoic and vitelline membranes were negative in both molecular biology and immunofluorescence assays (Fig 9). Notably, amplicons in positive specimens were obtained from viral RNA using either the specific primer to the viral genome, or the replicative intermediate, indicating active replication of viral RNA (Fig 9). We then sequenced the amplicons of these samples and compared them to the viral genome of the strain 17DD, finding 100% identity. Corroborating the specificity of this analysis, control animals and blocks without viral antigen (which were negative in the immunofluorescence microscopic studies) were all negative in the genomic viral RNA detection.

## Discussion

The YF vaccine is one of the most successful human vaccines ever made. Yet, until recently little was known about its mechanisms of immunity [29]. Similarly, although the 17DD vaccine has been produced in embryonated chicken eggs since 1937, the properties of viral proliferation in this model were poorly elucidated.

The present results show that in *Gallus gallus* embryos, the yellow fever 17DD virus is replicated in skeletal muscle cells (Fig 2), cardiomyocytes (Fig 3), renal tubular epithelium (Fig 5), lung parenchyma (Fig 6A and 6B), fibroblastoid cells of the connective tissues (Fig 6C and 6D), and in glial cells and neurons (Fig 4A and 4C). Our data also show that the skeletal muscle tissue has an important role in production of yellow fever 17DD viral particles, whereas no infection (including acidophilic corpuscles or steatosis) was detected in the liver. To clarify these findings and to test if the liver might become infected at a different stage, further studies should be conducted at different times of infection.

To emulate conditions used in YF vaccine manufacturing, we chose to work with 12-day-old chicken embryos inoculated with the YF vaccine virus and analyzed after 72 hours of infection [21,24]. While this increases the validity of the present study, in this stage, the events related to the normal embryo formation and the high rate of cell proliferation complicate the identification of histopathological changes putatively associated with the viral infection events. For example, the bone marrow formation starts and there is a high granulocytic and erythrocytic production in the yolk sac [30]. Because the immune system is still immature, the virus spreads and proliferates more easily [31–33]. The lung epithelium and gastrointestinal tract are under development, presenting cells in mitosis and apoptosis, as well as cells with cytoplasmic budding. To establish that these findings were not directly related to the infection it was necessary to study a sufficient number of embryos, and to use the sensitive immunostaining approach. As a result, we detected the sites of viral proliferation in a broad range of cell types from different tissues. Examining the positive cells with hematoxylin and eosin-stained serial sections allowed us to observe that some apoptotic cells were related to the viral infection (Fig 1).

Molecular tools were performed to corroborate the specificity of our results. Using RT-PCR we were able to detect viral genomic RNA in all immunofluorescence positive blocks (Fig 9). In contrast, the blocks where the infection was not detected by immunofluorescence were also negative to viral genomic RNA. Every PCR product was sequenced and compared with 17DD yellow fever virus reference genome. In samples where viral RNA was amplified, the presence of the replicative intermediate was evidenced, suggesting that viral replication is occurring where the virus protein was detected.

An intracellular pattern of viral antigen location could be clearly seen in infected cells (Figs 2B, 2D and 4C). We observed cells with intensive perinuclear labeling, consistent with the endoplasmic reticulum location, and a pattern suggesting cytoplasmic vesicular exocytosis. In muscular cells, the viral proteins detected follow the pattern of striations of the cytoskeleton consistent with the sarcoplasmic reticulum localization (Figs 2B, 2D, 7 and 8), showing the commitment of this organelle in virus production. Although the steps of cell interaction, assembly, and exit of the YF virus in the host cell are not fully understood, these mechanisms have been documented in other flaviviruses. The intracellular localization pattern we observed is consistent with that described for other flaviviruses, where the assembly of the viral particles happens in the surface of the endoplasmic reticulum, and their elimination is carried out by exocytosis [2,3,8,22,34,35].

Studies of wild yellow fever virus pathology in man, as well as other primates, and hamsters prove that the liver, kidneys and heart are the organs most affected by infection [3–7,36–38]. Fatal cases of vaccine-adverse diseases usually have the same histopathological findings as the wild YF infection [19,39,40]. Our results suggest that chicken livers are not affected by the infection. In man and other primates, Kupffer cells are the first infected cells in this organ, and for some authors, they constitute a barrier which protects hepatocytes from the yellow fever virus infection [3,8,11]. Although liver maturation is an early phenomena in chicken development [30], its embryos seem to have no Kupffer cells until the 13th development day [41], so the absence of these cells could be a bottle neck to virus infection in this organ. Our results don’t lead us to reach a definitive explanation for this absence of liver infection, but it is possible that Kupffer cells are actually a dissemination component of the virus in the liver and that the absence of these cells in chicken embryos, in this stage of development, may be one of the reasons why in this model the liver is not infected. Extrapolation of these data to other animal models could break the paradigm of Kupffer cell function during YFV infection, making these cells a spreading, not a protective, component. On the other hand, liver infection could be a subsequent event in YFV infection, so it is necessary to investigate later times of infection, to confirm this data.

The kidneys were positive in the tubular epithelium without apparent involvement of the glomerulus (Fig 5). The infection of the kidney tubular epithelium cells by yellow fever virus has been demonstrated in man and Rhesus monkeys, who, unlike our chicken embryos, also experienced renal failure [8,42]. Although some cells undergoing apoptosis were found in the same areas where viral antigens were detected (Fig 1C), apparently the *Gallus gallus* kidney damage is mild relative to severe cases in man. It was interesting to find the virus in the lumen of the renal tubular epithelium (Fig 5B and 5C), because others have found the vaccinal 17DD virus in the urine of vaccinated patients [43].

We also detected the virus and apoptotic cells in the myocardium of infected animals (Fig 3). The presence of virus in the heart has been identified in humans, where viral antigens are found in the myocardium of infected patients, with a necroapoptotic and steatosis profiles similar to what is observed in the kidneys and liver [8,42]. In the present model, we did not find any steatosis, and although apoptotic figures were seen in the affected areas, there was no associated necrosis.

In this study, infected skeletal muscle cells were found throughout the bodies of the embryos. Because of its large area and the observed intensity of infection, this tissue could be the major site of viral replication. Since no other work on the pathology of YF infection mentions skeletal muscle infection, this finding is unprecedented. Yet, our results corroborate Fox and Laemmert’s [44] identification of higher viral titers in muscle and nerve tissues after 72h of infection. Although no other data on YF skeletal muscle infection are available, clinically it is known that one of the main symptoms of yellow fever is muscle pain, in addition to the significant increase of aminotransferases in critically ill patients, which can be partially explained by the cytopathic effect in cardiac and skeletal muscle [3,4]. Although it is necessary to examine this phenomenon in humans, infection of these cells (and the consequent rhabdomyolysis observed) could justify these symptoms. Conversely, other arboviruses associated with symptoms of muscular pain similar to those seen in YF, such as Chikungunya [45], Mayaro [46], and Ross River [47] viruses, can infect muscle cells in humans and in experimental models.

In *Gallus gallus*, desmin expression is not restricted to skeletal muscle cells, but also occurs in mesenchymal-like cells. The anti-desmin antibody helps to detect skeletal muscle cells in different regions of the animal, and allows the disclosure of mesenchymal infected cells in the lung parenchyma (Fig 6A and 6B). Different tissues also showed rare infected fibroblastoid cells (Fig 6). The infection of fibroblasts in culture is already known [48], but apparently the susceptibility to infection of these cells *in vivo* seems less expressive than *in vitro*, at least at 72 hours post infection. Although the yolk sac is the site of virus inoculation, this extraembryonic membrane showed no evidence of infection. Only fibroblastoid cells in the muscular layer of the yolk stalk were positive (Fig 6F). Likewise, molecular detection techniques also showed the chorioallantoic membrane was negative. Our results do not corroborate Fox and Laemmert’s detection of virus titer in extracts of these membranes [44].

The 17DD strain maintains some neurovirulence, observed when a virus sample is intracerebrally inoculated in mice and non-human primates. In addition, rare cases of vaccine-associated neurotropic disease (YEL-AND) have been documented in children younger than 9 months, mainly due to the immaturity of the blood–brain barrier. Patients with immunodeficiency also show documented YEL-AND cases [1,8,29]. In this study, we observed animals with infection in the brain (Fig 4A and 4C), cerebellum, and spinal cord cells (Fig 4B), probably also due to immaturity of the blood–brain barrier, which is formed in chicken embryos after 15 days of development [49].

The lack of inflammation in the affected tissues could be due to the immature immune system of these animals [31–33], but it may also be related to characteristics of YFV. In affected tissues, infected cells were either isolated or present in small clusters, suggesting that the infection is mild in most of these tissues. Overall, there was great variability in response and susceptibility to infection among our sample, possibly because *Gallus gallus* is not an isogenic animal. Yet, despite the YF 17DD infection, most of the animals, when left under favorable conditions, are born without any sequelae of the disease [44].

In conclusion, our data suggest that YF 17DD infection of *Gallus gallus* embryos is mild but systemic, and affects various tissues and cells with different embryonic origins; however, not all cells are susceptible to virus infection. The skeletal muscle tissue seems to be the main site of production of the YF 17DD virus due to its large body area and the intensity of its cell labeling.

The elucidation of cell and tissue competence could help develop new possibilities for producing the YF 17D virus (e.g., in cell cultures). This approach could, in turn, minimize problems related to high levels of chicken proteins in the vaccine [48,50]. Our data are particularly relevant to this problem because recent studies have suggested the possibility of using virus 17DD as another vaccine production platform, in which 17DD has proved to be an effective viral vector to recombinant proteins of other flaviviruses (including Japanese encephalitis, West Nile, and dengue viruses), and of other unrelated organisms, such as *Plasmodium yoelli* and *Trypanosoma cruzi* [51–53]. Nevertheless, further studies are needed to better elucidate YFV infection, and to establish how it occurs in humans. Of particular interest, kinetic studies should seek to clarify, for example, when and how viral particles reach the organs and tissues identified in the present work.
